# An Optimized Instance Segmentation of Underlying Surface in Low-Altitude TIR Sensing Images for Enhancing the Calculation of LSTs

**DOI:** 10.3390/s24092937

**Published:** 2024-05-05

**Authors:** Yafei Wu, Chao He, Yao Shan, Shuai Zhao, Shunhua Zhou

**Affiliations:** 1 The Key Laboratory of Road and Traffic Engineering, Ministry of Education, Tongji University, Shanghai 201804, China; 1710514@tongji.edu.cn (Y.W.); hec_tjjt@tongji.edu.cn (C.H.); shanyao@tongji.edu.cn (Y.S.); 2 Shanghai Key Laboratory of Rail Infrastructure Durability and System Safety, Tongji University, Shanghai 201804, China; 3 Department of Civil and Environmental Engineering, Hong Kong Polytechnic University, Hong Kong, China

**Keywords:** low-altitude TIR sensing image, urban rail transit hub, instance segmentation, deep learning, underlying surface

## Abstract

The calculation of land surface temperatures (LSTs) via low-altitude thermal infrared remote (TIR) sensing images at a block scale is gaining attention. However, the accurate calculation of LSTs requires a precise determination of the range of various underlying surfaces in the TIR images, and existing approaches face challenges in effectively segmenting the underlying surfaces in the TIR images. To address this challenge, this study proposes a deep learning (DL) methodology to complete the instance segmentation and quantification of underlying surfaces through the low-altitude TIR image dataset. Mask region-based convolutional neural networks were utilized for pixel-level classification and segmentation with an image dataset of 1350 annotated TIR images of an urban rail transit hub with a complex distribution of underlying surfaces. Subsequently, the hyper-parameters and architecture were optimized for the precise classification of the underlying surfaces. The algorithms were validated using 150 new TIR images, and four evaluation indictors demonstrated that the optimized algorithm outperformed the other algorithms. High-quality segmented masks of the underlying surfaces were generated, and the area of each instance was obtained by counting the true-positive pixels with values of 1. This research promotes the accurate calculation of LSTs based on the low-altitude TIR sensing images.

## 1. Introduction

With rapid urbanization, natural underlying surfaces have been changed tremendously by the construction of artificial surfaces such as asphalt and concrete pavements. In particular, rail transit plays a profound role in urbanization, especially under the transit-oriented development (TOD) model [1]. Urban rail transit hubs (URTHs), which are crucial nodes in the urban rail transit network, dramatically change the land type and land cover [2]. Moreover, the thermophysical properties of the artificial underlying surfaces changed the thermal environment significantly [3,4,5,6]. Consequently, energy budgeting has been altered, and the land surface temperatures (LSTs) of the built environment have been elevated [7], which contributes to the heat island effect [8] and various environmental issues [9,10,11,12].

Interest in the study of metropolitan thermal landscapes has increased rapidly, especially at the scale of city blocks, commonly regarded as the bedrock of cities, which present microcosmic interactions that cumulatively determine broader urban dynamics. A major focal point within this field of study is the quantitative inversion of LSTs [13], which has been highlighted by numerous scholars for its implications in revealing the differences in surface temperature across urban terrains [13,14]. The applications of LSTs in various purposes emphasizes the importance of the quantitative inversion of LSTs, such as monitoring the natural calamities [15], assessing the balance of urban-ecological systems [16], and evaluating the patterns of landscape [17] and the urban heat island phenomena [18].

Understanding the temporal and spatial distribution characteristics of LSTs is beneficial for improving our understanding of micro-urban thermal dynamics. Various algorithms have been proposed to retrieve LSTs using a Landsat thermal-infrared dataset [19], thermal band data of the Landsat Thematic Mapper [20,21], and high-resolution radiometer data [22,23,24]. These algorithms have been proven to be extremely effective. In addition, the estimation of LSTs using low-altitude thermal infrared remote (TIR) sensing images has become more popular recently because of the flourishing development of unmanned aerial vehicle (UAV) techniques. For instance, the authors previously proposed a practical and feasible method for estimating LSTs at the block scale using near-ground meteorological data and low-altitude TIR sensing images acquired using UAV [25]. Although the proposed method is proficient at obtaining the LSTs of individual points on the underlying surface, it cannot estimate LSTs for distinct types of underlying surfaces within the context of complex underlying surfaces. To address this limitation, it is crucial to recognize and segment the various types of underlying surfaces accurately before estimating LSTs. In practice, TIR sensing images extend through a variety of regions covered by different types of underlying surfaces. And it is challenging to recognize and segment these various types of underlying surfaces in the images because of the influence of distractors having characteristics similar to those of the underlying surfaces. Therefore, it is highly necessary to develop approaches capable of achieving the accurate recognition and segmentation of underlying surfaces to be able to estimate LSTs for specific surface types precisely, which in turn will help researchers develop a comprehensive understanding of the urban micro thermal environment.

Deep learning (DL) has attracted significant attention because of its capabilities in object recognition and segmentation. The back-propagation algorithm for DL proposed by LeCun et al. [26] enables the automatic discovery of intricate structures in large image datasets. The convolutional neural network (CNN), a DL algorithm, is specifically designed to process images that are represented as multiple arrays [27,28]. Numerous applications of CNNs in the tasks of object detection and segmentation of various kinds of applications have proved to be effective and reliable [27,29,30,31,32,33,34,35,36]. Moreover, one of the key breakthroughs in this field was a mask region-based convolutional neural network (Mask R-CNN) algorithm [37] for processing the task of object instance segmentation. The instance segmentation comprises two major tasks, i.e., the detection and semantic segmentation of objects. The Mask R-CNN algorithm detects the objects in various kinds of images efficiently and simultaneously generates the segmentation masks with high quality for each instance. Applications of the Mask R-CNN algorithm for processing images have led to significant advancements, particularly in the fields of AI monitoring and environment exploration [38]. Additionally, the Mask R-CNN algorithm has been employed to delineate and classify ice-wedge polygons, helping researchers understand polar landscapes [39]. Furthermore, the applications in ship detection [40,41], infrared image processing tasks for power equipment [42], and the instance segmentation of moisture marks in shield tunnel linings [43,44] have been proved available.

However, the application of the deep CNN framework in object detection and segmentation in the low-altitude TIR sensing images of underlying surface has seldom been reported because of the complex distribution of underlying surfaces. In the realm of TIR sensing, where complex backgrounds exist and obtaining accurate surface area information poses challenges, there has been a limited application of deep CNNs. Additionally, obtaining accurate geometric area information for different underlying surfaces in the low-altitude TIR sensing images is still a challenge. As mentioned previously, the Mask R-CNN algorithm has already shown prowess in various fields of image instance segmentation. Therefore, this study sought to extend its capabilities to segment the low-altitude TIR sensing images to mitigate the effect of complex backgrounds on the segmentation of underlying surfaces and their area computation.

The goal was to introduce an instance segmentation framework to solve the aforementioned obstacles in the calculation of LSTs of each type of the underlying surface in the context of complex underlying surfaces. To achieve this goal, a Mask R-CNN algorithm was introduced and optimized to meet the demands of the unique application environment, that is, the URTH. We first established an image dataset comprising 1500 low-altitude TIR sensing images depicting markings on the underlying surfaces. The low-altitude TIR sensing image dataset contained different types of underlying surface marks and was annotated using the LabelMe tool (Version 4.5.13). Once the image dataset was meticulously prepared, the optimization of the Mask R-CNN algorithm was performed through three crucial processes, namely, the extraction of features, the generation of region proposals, and the identification of underlying surface marks. To evaluate the performance of both the original and the structurally optimized Mask R-CNN algorithms, comprehensive metrics, namely, the mean average precision (m*AP*) [37], *F*_1_ score [45], intersection over union (IoU) [27], and inference time, were used with 150 new testing TIR images. The structural-optimized MASK R-CNN algorithm identifies the types of the underlying surfaces and extracts the areas of the underlying surfaces in the low-altitude TIR sensing images more effectively and accurately. These identification and area data can provide a foundation for further precise calculation, distribution, and analysis of the LSTs based on the low-altitude TIR sensing images.

## 2. TIR Sensing Image Dataset of the Underlying Surfaces at an Urban Rail Transit Hub

Using DL to complete the task of instance segmentation of the underlying surface of a low-altitude TIR sensing image relies on the quantity of well-labelled and annotated image data as well as the computation power. The problem of acquiring an extensive amount of well-labelled image data has been resolved through the continuous advancement of labelling techniques. However, the limited availability of a well-annotated and openly accessible dataset that includes detailed information about the markings on the underlying surfaces of URTHs is currently worth noting. Therefore, obtaining a sufficient number of low-altitude TIR images that contain underlying surface marks is of utmost importance to the instance segmentation of the underlying surfaces of URTHs.

### 2.1. Description of the Urban Rail Transit Hub

**Figure 1:**
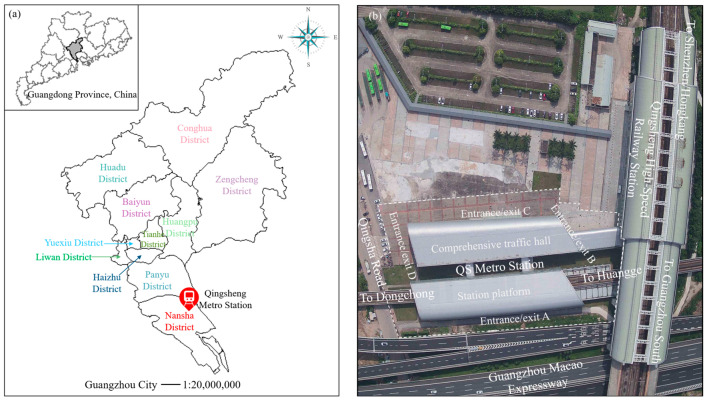
Overview of the QS hub station: (**a**) location of the QS hub station and (**b**) layout diagram and surroundings of the QS hub station.

Specific criteria must be satisfied to collect low-altitude TIR sensing image data from URTHs. These criteria include the multiple types of underlying surfaces at a station, noticeable differences in the regions of these underlying surfaces, and the distribution of these underlying surfaces being relatively complex, which meets the requirements of our research work. Additionally, as one of the research cooperation units, the Guangzhou Metro Group can provide the permits for the UAV flights. The Qingsheng (QS) station, a hub station of Guangzhou Metro Line 4, aligns perfectly with these requirements for obtaining low-altitude TIR sensing images. Thus, the QS station, located on Qingsha Road, Nansha District, Guangzhou, a megacity in South China, was chosen for the imaging task, as depicted in Figure 1a [46].

The QS hub station serves a central function in the transportation network of the Guangzhou Metro. Serving as both an elevated station and a transfer point, the QS hub station connects the Guangzhou Metro Line 4 and the future Line 18 with Guangzhou, Shenzhen, and the Hong Kong high-speed railway line, as illustrated in Figure 1b. Additionally, to accommodate different passenger flows and directions, the station was equipped with four distinct entrances and exits (Figure 1b).

**Figure 2:**
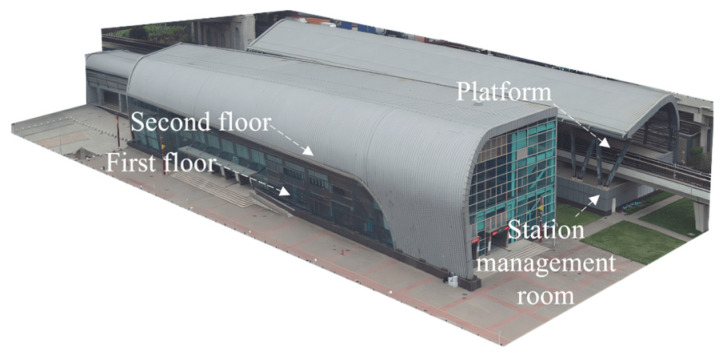
Photographs of the layout of the QS hub station: the two buildings and related facilities.

**Figure 3:**
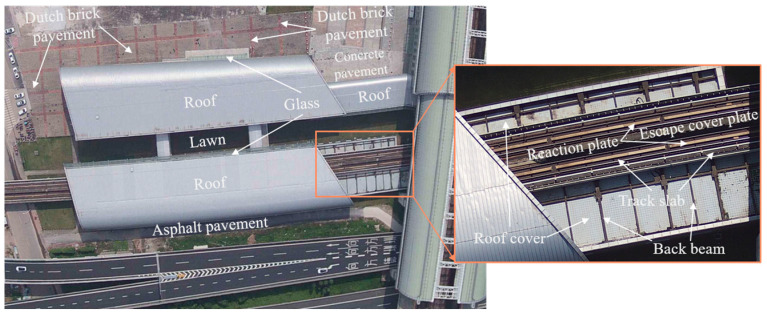
Types of underlying surfaces of the QS station.

Architecturally, the station is designed with two two-story structures (Figure 2) to ensure that it can accommodate a significant number of passengers and streamline their movement. The QS hub station exhibits a diverse range of underlying surfaces with a notably intricate distribution, as depicted in Figure 3. As shown in Figure 3, the QS hub station has 11 types of underlying surfaces, including various types of specific underlying surfaces in the urban rail transit area. In addition, there are significant differences in the area of various types of the underlying surfaces, which pose significant challenges for the task of instance segmentation in the low-altitude TIR images. The types of underlying surfaces at the QS hub station are listed in Table 1.

### 2.2. Acquisition of the Low-Altitude TIR Sensing Images

**Figure 4:**
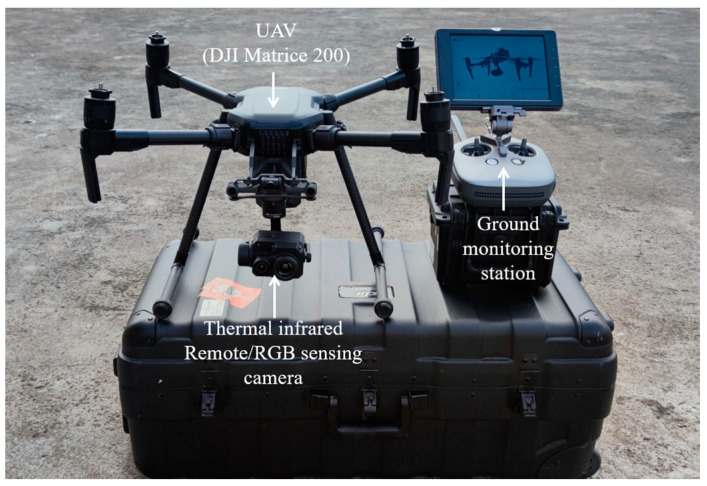
UAV platform and low-altitude TIR imaging sensors.

A low-altitude TIR sensing system, illustrated in Figure 4 [25], was employed to acquire the low-altitude TIR sensing images of the underlying surfaces at the QS hub station.

The low-altitude TIR sensing system consisted of a TIR pictorial device along with an RGB camera, integrated onto a DJI Matrice 200 unmanned aerial vehicle (UAV). The parameters of the TIR sensing system, according to Wu et al. (2022) [25].

Several crucial factors were considered to ensure comprehensive coverage of the low-altitude TIR sensing images captured using the TIR sensor, and a specific flight setup was designed to ensure compliance with the standards stipulated in GB/T 39612–2020 of the Ministry of Natural Resources of China [47]. Utilizing the FOV (field of view) of the TIR camera’s wide-angle lens, parameters such as a 75% offset distance and overlapping rates for both the flight routes and belt were established. This meticulous planning ensured optimal data capture. Furthermore, the UAV was operated at the low altitude of 150 m at a speed of 10 m/s, thereby guaranteeing the required spatiotemporal resolution. By considering these factors, we ensured comprehensive coverage of the TIR images while meeting the requirements of the standard. This work is significantly important for applications in the geographical information and resource management fields.

**Figure 5:**
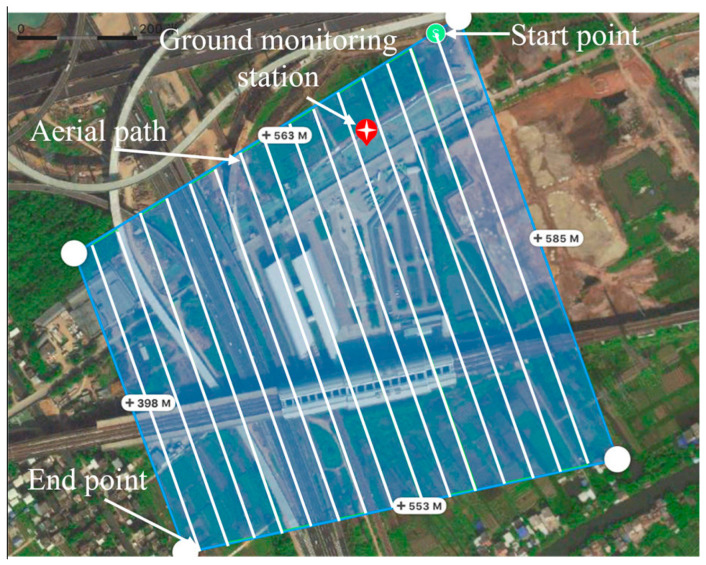
Aerial path plan of the low-altitude image system.

**Figure 6:**
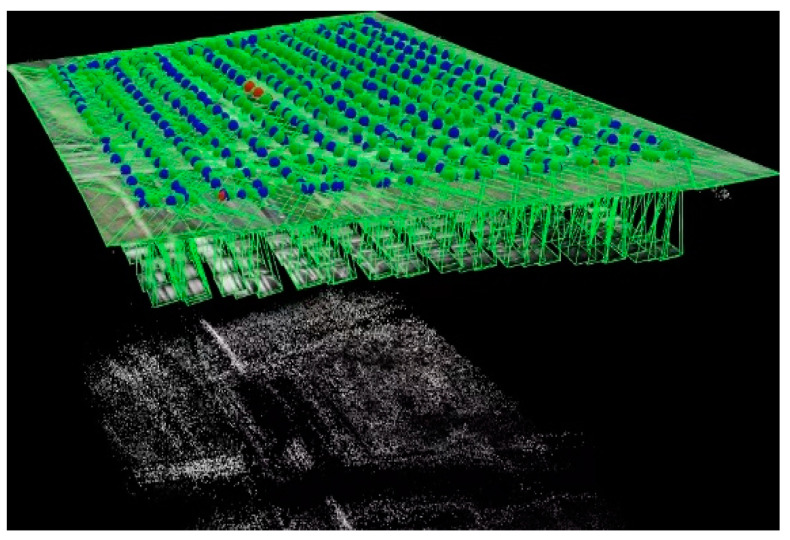
Map of the point cloud (Blue dots represent the TIR sensing images; Green dots represent the RGB images; Red dots indicate the image quality are poor.).

The passage included 15 flight routes, encompassing a total of 30 flight points. These routes varied in length, with the longest measuring 585 m and the shortest measuring 398 m. The spacing between routes was 27.5 m (Figure 5). The combined distance of all flight routes was 7927 m. Visual representations of the flight points are illustrated in the point-cloud diagram in Figure 6.

The UAV followed a predetermined flight plan after being launched from the starting point, as shown in Figure 5. It ascended vertically to reach the predetermined altitude and then proceeded to cruise automatically based on preset instructions from the ground controller, as shown in Figure 4. Upon completing the scheduled route, the UAV returned to its take-off point.

Furthermore, to attain a high spatiotemporal resolution of the TIR sensing image data and ensure the capture of an adequate number of images during each flight, we configured an automatic shooting time interval of 3 s. Each flight had an approximate duration of 20 min, which provided comprehensive coverage of the designated area. Flight operations commenced in June 2019 and continued until March 2021. However, due to the impact of the COVID-19 pandemic, the flight activities were temporarily suspended during this period. Throughout the duration of the flights, fine and stable weather conditions were required to ensure the optimal data collection. A total of 964 low-altitude TIR images was obtained, as documented in Table 2.

### 2.3. Establishment of Low-Altitude TIR Sensing Image Dataset

The low-altitude TIR sensing images obtained during the flights were initially stored at a resolution of 640 × 512 pixels on the flash memory card of each flight. To ensure the reliability and accuracy of the TIR sensing image data, a stitching process was performed to obtain images with a higher resolution of 910 × 512 pixels. These stitched TIR sensing images were then cropped to a resolution of 720 × 512 pixels, and images containing marks of the underlying surfaces were selected for further analysis. To align the image size with the dimensions of the markings on the underlying surfaces, we further cropped the selected images into two scales with the distinct resolutions of 640 × 512 and 720 × 512 pixels, respectively.

**Figure 7:**
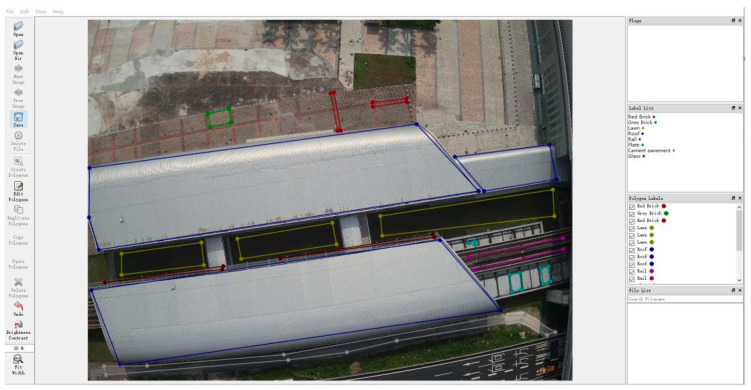
Marks of the underlying surfaces annotated using LabelMe.

Subsequently, all the chosen TIR sensing images were annotated using LabelMe. The markings on the underlying surfaces were manually annotated by creating polygons to delineate their boundaries, as illustrated in Figure 7. Once the polygons had been drawn, labels were assigned in the dialogue and recorded in the corresponding JSON file.

**Figure 8:**
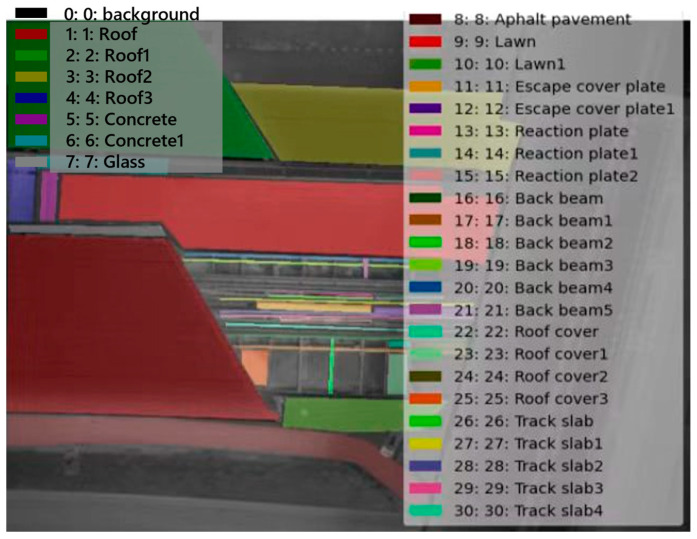
Illustration of the annotated low-altitude TIR sensing images.

After completing the annotation process, a detailed file capturing the coordinates of various markings was created. To ensure the compatibility with popular machine learning tools, the file was then transformed into the format which is similar to the Microsoft COCO dataset [48] utilizing Python 3.6 for the transformation. The low-altitude TIR sensing image dataset consisted of 1500 images and their corresponding JSON files, which stored the geometric information of the underlying surface marks. A portion of the annotated low-altitude TIR sensing images is shown in Figure 8.

The dataset of the low-altitude TIR sensing images was separated into three distinct parts for the training, validation, and testing. In accordance with the methodology outlined by Shahin et al. (2004) [49], 90% of the dataset, which comprised 1350 images, was randomly selected for calibration purposes, leaving the remaining 10% of the dataset, which comprised 150 images, for testing.

**Figure 9:**
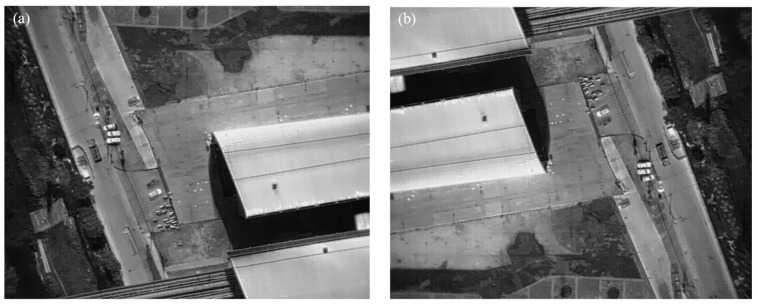
Image data enhancement: (**a**) raw image and (**b**) vertically flipped image for data augmentation.

The calibration data were subsequently split, with 80% (1080 images) allocated for training the algorithm and the remaining 20% (270 images) reserved for the validation to assess the performance of the algorithms. To augment the dataset of the images, the two algorithms automatically employed vertical flipping for both the datasets of training and validation, as optimized in Figure 9.

### 2.4. Unique Characteristics of the Underlying Surfaces of the URTH

The distinctive characteristics of the markings on the underlying surfaces of the urban rail transit hub, including aspects such as color, texture, and boundary information, are pivotal for effective object detection and recognition. These attributes differentiate the markings on the underlying surfaces of the urban rail transit hub from those on other types of underlying surfaces. The digital number (DN) values in the low-altitude TIR sensing images represent the darkness of the underlying surfaces. The different types of underlying surfaces exhibit distinct DN values, enabling their differentiation. The texture of the underlying surfaces is another significant indicator useful for distinguishing different surfaces in TIR sensing images. Variations in texture characteristics between different surfaces are significant and aid identification. Furthermore, the area occupied by the different underlying surfaces also differs significantly, which provides an additional cue for detecting and recognizing different marks in the low-altitude TIR sensing images. The significant feature disparities in the underlying surfaces within the URTH, as highlighted in the low-altitude TIR sensing images, present unique features. To address this, the Mask R-CNN algorithm was introduced, enabling the accurate detection and recognition of the objects in this specific environment and ensuring enhanced operational efficiency and accuracy.

The Mask R-CNN algorithm stands out as a pivotal innovation in the realm of computer vision. Exceptionally skilled at pinpointing and distinguishing various marks within images, Mask R-CNN accesses the essence of images consisting of arrays of pixel values. The convolutional layers within the algorithm harness this intrinsic nature to achieve remarkable precision. By analyzing the DN values of neighboring pixels, these layers are adept at discerning the boundaries and edges of marks. This level of spatial accuracy is remarkable in itself; however, the algorithm does even more. The subsequent convolutional layers go beyond mere boundaries; they extend into patterns and motifs. They can identify the precise configurations of edges and even discern the subtle textural features of the marks, allowing Mask R-CNN to provide an unparalleled intricate understanding of the underlying surfaces. This multi-layered approach empowers it to excel in tasks ranging from image segmentation to object detection and scene comprehension, making it a versatile and invaluable tool in the realm of computer vision.

The subsequent convolutional layers within the Mask R-CNN algorithm are tasked with assembling the patterns identified into more comprehensive compositions that correspond to distinct sections of the markings on the underlying surfaces. This process enhances the ability to capture intricate details about the markings and ultimately aids in achieving accurate recognition. By learning from the dataset through a self-teaching procedure, the Mask R-CNN algorithm effectively extracts and interprets the distinctive features of the underlying surface marks. The feature learning enables the algorithm to describe a raw image in terms of the specific parts related to the marks on the underlying surfaces. Once the Mask R-CNN algorithm has learned the features, it is capable of recognizing and segmenting the marks of the underlying surfaces from the background of a low-altitude TIR sensing image. The segmentation is achieved even in the presence of distractors, such as other underlying surfaces that may exist outside the URTH.

The core concept of the Mask R-CNN is its capacity to learn and extract deep features from data autonomously, particularly in the context of low-altitude TIR sensing images. This enables the model to delineate underlying surface marks accurately, even when they are surrounded by distracting elements, such as regions outside urban rail transit hubs. In essence, the strength of the Mask R-CNN lies in its ability to discern and segment critical surface features amid complex backgrounds.

## 3. Framework for the Instance Segmentation of the Low-Altitude TIR Sensing Images

**Figure 10:**
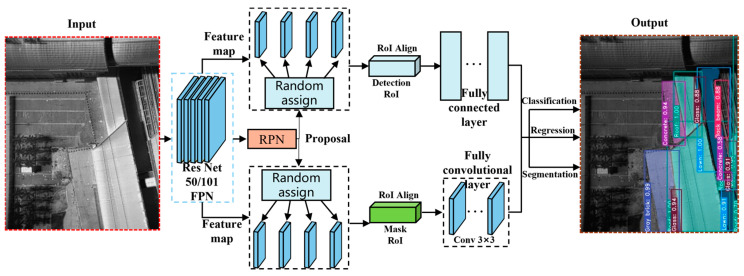
Framework details of the original Mask R-CNN algorithm.

To identify markings on the underlying surfaces accurately and simultaneously create high-quality segmentation masks within low-altitude TIR sensing images, a structure optimized Mask R-CNN algorithm was proposed. The original algorithm [37] combines a Faster R-CNN according to Ren et al. (2017) [50] to perform the classifications and regression tasks of bounding-box with an FCN branch for predicting the segmental masks, as depicted in Figure 10.

The original algorithm progresses through three main stages: (1) the extractions of the features at different depths of the underlying surface marks of an input image with a backbone architecture; (2) the generations of the proposals of underlying surface marks with a region proposal network, i.e., RPN; and (3) the classification, recognition of bounding-box, and prediction of the masks of underlying surfaces through a head architecture, as depicted in Figure 10.

### 3.1. Backbone Architecture of the Feature Extraction of Underlying Surface Marks

**Figure 11:**
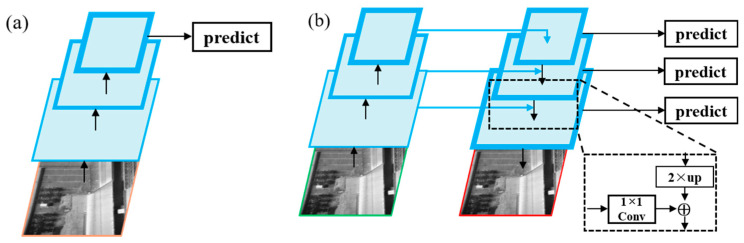
Feature pyramid: (**a**) single map and (**b**) feature pyramid network (Arrows represent the flow of data).

Typically, the backbone architecture employs a deep CNN to calculate the feature hierarchies of the underlying surface marks within TIR sensing images layer by layer iteratively. However, the CNN’s feature hierarchy calculates feature maps with different depths and large semantic gaps between the different levels of the feature maps, which is a result of the distinct depths of the convolutional layers. Higher resolution feature maps, capturing fine-grained details, include low-level features; whereas, feature maps with a lower resolution, representing more abstract information, contain high-level features. Recent investigations concerning the recognition of marks [32,34,51] have utilized the CNNs to generate a single coarser-resolution feature map with high level, where the marks are predicted, as shown in Figure 11a.

Nevertheless, this practice cannot benefit from the potential advantages associated with integrating higher-resolution feature maps within the feature hierarchy. The omission becomes particularly problematic in the recognition of small objects. Because of the pooling operations applied by CNNs to the ultimate layer, a potential peril of forfeiting crucial low-level features exists. This, in turn, renders the task of precisely detecting and discriminating smaller objects on the underlying surfaces notably arduous. Therefore, to recognize small marks of the underlying surfaces effectively, it is extremely important to maintain the higher-resolution feature maps that possess information of low-level features because this practice enables a more comprehensive representation and understanding of the intricate details related to small objects or marks.

Feature pyramid networks (FPNs), which was proposed by Lin et al. (2017) [52], provide an effective remedy for addressing the difficulties encountered in computer vision, particularly within the context of applying the Mask R-CNN algorithm to low-altitude TIR sensing imagery.

FPNs leverage the inherent pyramidal structure of a CNN’s feature hierarchy to maintain strong semantics across all scales, while also effectively providing representational power, computational efficiency, and acceptable memory consumption. Therefore, based on the algorithm, FPNs serve as the backbone architecture, enabling the extractions of essential features from the underlying surface marks in low-altitude TIR images. The FPN accomplishes feature fusion through a top-down method and lateral connections which connects the high-resolution but semantically weak features with the low-resolution but semantically strong features (Figure 11b). Overall, leveraging the FPN as the backbone architecture of the algorithm facilitates the processing of the hierarchical features presented in the feature pyramid structure, thereby offering an effective solution to the challenges in computer vision applications, especially in the domain of low-altitude TIR sensing.

**Figure 12:**
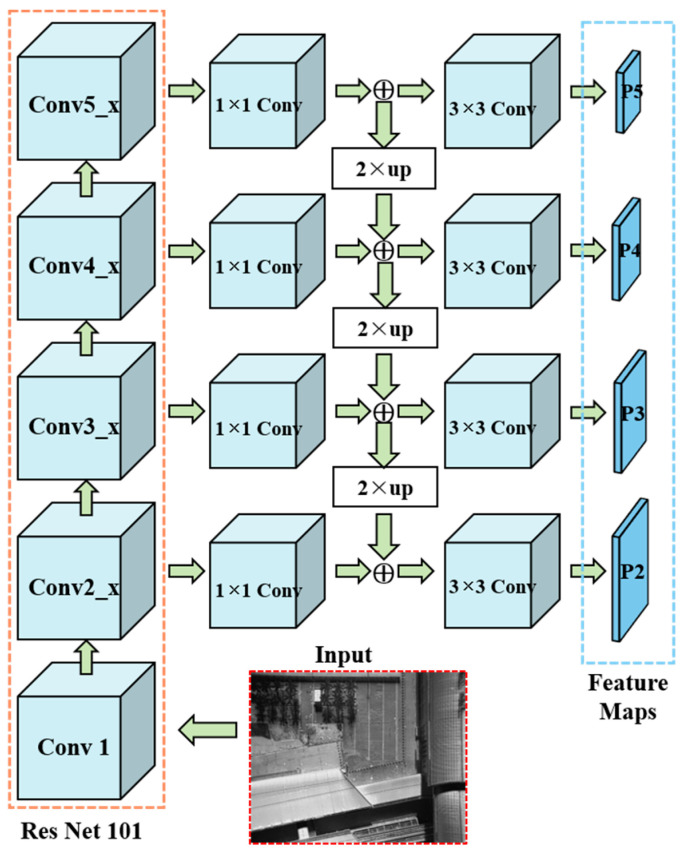
Architecture of an FPN.

Figure 12 shows the architecture of an FPN. To construct the feature pyramid, a conv5_x layer undergoes a transformation through a 1 × 1 convolutional layer, resulting in the creation of the coarsest-resolution feature map. The initial map captures the most semantically significant features related to the underlying surface marks. Subsequently, a two-fold spatial resolution up-sampling is performed, followed by a merging with lower-resolution maps through element-wise addition. The iterative progression continues, yielding finer-resolution feature maps at varying levels and ultimately culminating in the generation of a series of feature maps, which were denoted as P2 to P5. Each of these feature maps is equipped with a 3 × 3 convolutional layer for further up-sampling. Importantly, these feature maps serve as critical tools for independently conducting predictions related to the underlying surface marks at different levels, enhancing the efficiency and accuracy of various computer vision tasks, as exemplified in Figure 12.

### 3.2. Generation of the Region Proposals for Underlying Surface Marks with an RPN

**Figure 13:**
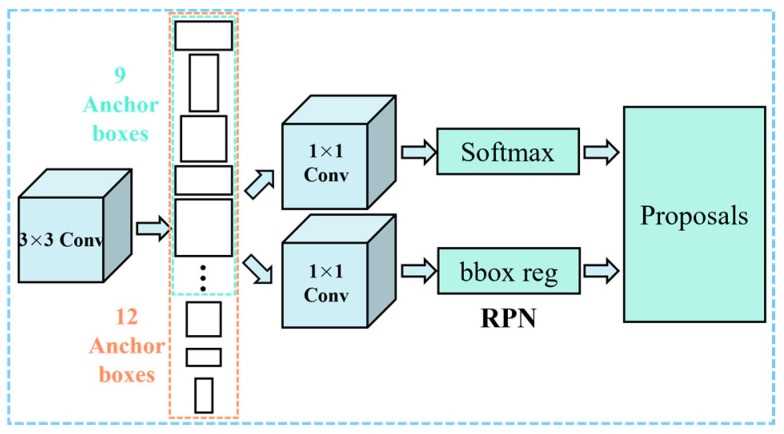
RPN of the Faster R-CNN with a traditional CNN backbone architecture.

Object detection holds a crucial position within the expansive domain of computer vision. Object detection networks predominantly rely on the regional proposal algorithms to hypothesize the coordinates of objects. These algorithms suggest potential locations where objects might be present in an image. However, one major drawback is the intensive computation required for these proposals, which significantly hinders the network’s efficiency in terms of runtime. In response to this challenge, researchers introduced region proposal networks (RPNs) to address the problem of region proposal computation by sharing extracted features with previously mentioned backbone architecture, which makes the process nearly cost-free. The sub-network employs a dense 3 × 3 sliding window that traverses a feature map produced by the backbone to conduct dual tasks: object or non-object binary classification, and the bounding-box regression. Notably, for each position of the sliding window, the RPN predicts a variety of anchor scales based on the given aspect ratios. The classification and regression tasks are realized by a 3 × 3 convolutional layer, following two 1 × 1 sibling convolutions for the classification and regression, as exemplified in Figure 13.

Notably, at each location of the sliding window, anchors with three scales (1:2, 1:1, and 2:1) and three aspect ratios (1:2, 1:1, and 2:1) are predicted simultaneously, as shown in Figure 13 (blue dashed box). The anchors not only serve as a reference for classification and regression but also enable the RPN to produce refined region proposals. These proposals are then meticulously processed in the subsequent stages of the object detection system, ensuring the accurate and efficient identification of objects.

In the analysis of low-altitude TIR sensing images, the FPN plays a pivotal role in extracting the nuances of the underlying surface marks. The generations of the feature maps at four distinct scales, namely the P2 to P5 (Figure 12), guarantee a comprehensive and intricate representation. Moreover, incorporating three anchors with distinct aspect ratios at each level culminates in a comprehensive set of 12 anchors throughout the pyramid, as shown in Figure 13 (orange dashed box).

During the initial training phase, each FPN level yields 2000 proposals. However, these proposals frequently overlap, leading to redundancies. To address the redundancy problem, the non-maximum suppression (NMS) according to Ren et al. (2017) [51], which filters proposals based on the classification scores, is employed. Subsequently, the procedure fine-tunes the proposals, retaining only the foremost 256 proposals with the highest rankings to be subsequently employed for the functions following the branches of the tasks, i.e., the classification, bounding-box regression, and segmentation.

### 3.3. Underlying Surface Mark Identification with the Head Architecture

**Figure 14:**
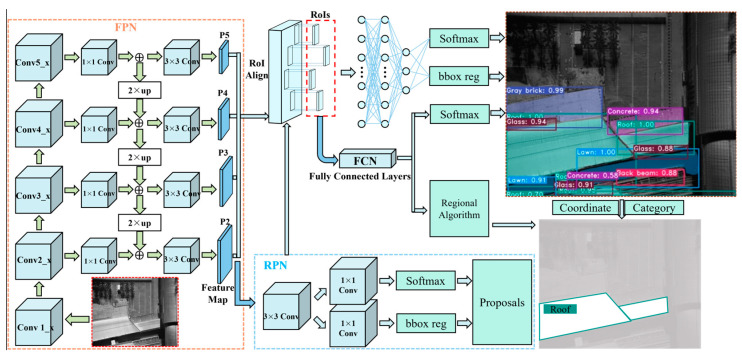
Overall structure of the optimized Mask R-CNN algorithm.

The visual representation in Figure 14 indicates the intricate architecture of the structure-optimized algorithm, which is an advanced model tailored to image processing tasks. Three pivotal components are central to its functionality: the backbone structure, known as the FPN, the RPN, and the all-encompassing head architecture. Serving as the initial gateway, the FPN ingests the complete image through a series of convolutional and max-pooling operations and generates feature maps spanning multiple scales. These feature maps become the foundation upon which the RPN crafts proposals, pinpointing potential surface marks within the image. Of these myriad proposals, a selected top 256, based on the distinguishing scores, are ushered into the region of interest (RoI) align layer according to He et al. (2017) [37]. Here, each proposal is transformed into a precise feature vector, which subsequently navigates through the head architecture undergoing classification and bounding-box regression. Notably, in a parallel operation, an innovative mask branch diligently crafts segmentation masks of impeccable quality for every RoI. The seamless fusion of the backbone, RPN, and head architecture, equipped with separate branches of the tasks, i.e., the classification, bounding-box regression, and segmentation, endows the optimized Mask R-CNN with the capability to excel in the precise detection and segmentation of surface marks, revealing the subtle details within the input images.

The structure of the Mask R-CNN algorithm was further developed by adding a branch of a regional algorithm, which can calculate the area of a certain type of underlying surface. Specifically, the regional algorithm is placed next to the FCN head branch (Figure 14), and it can assign a value of 1 to pixels of the underlying surface regions that are segmented by the FCN head branch. Therefore, the geometric area of the underlying surface can be obtained by counting the number of the pixels with value of 1. And, the FCN head branch can obtain the roof region through judging whether a certain pixel was the type of the roof or other types of underlying surface, as shown in Figure 14. Then, the regional algorithm is activated to compute the geometric area of the roof by counting the number of the pixels of the obtained roof region. In addition, from a statistical analysis perspective, the calculation of the area of a certain type of underlying surface also facilitates analysis of the average LSTs of the various kinds of the underlying surface.

### 3.4. Training the Mask R-CNN Algorithms

Training the two Mask R-CNN algorithms for the instance segmentation of the marks of the underlying surface within the low-altitude TIR sensing images, practices were carried out adopting a computer equipped with an Intel Core i7-10700F central processing unit (CPU), two NVIDIA GeForce RTX 3060 graphics processing units (GPUs), and 64 GB of random access memory (RAM), each with 16 GB of graphics memory. The two algorithms were actualized based on Tensor Flow-GPU = 2.4.0, a software system developed by Google Brain. The environment of the software framework was implemented using Python 3.8, Anaconda 3.0, CUDA 11.0, and the corresponding cuDNN (version 8.0.5).

For the two Mask R-CNN algorithms, an input raw image underwent resizing, ensuring that its shorter side measured 512 pixels in length. To train both algorithms on the two GPUs, synchronous stochastic gradient descent (SGD) proposed by Le Cun et al. (1998) [26] was utilized. Each mini batch processed two raw TIR images per GPU and the 256 RoIs of each image. Momentum and initial weight decay were configured at 0.9 and 1 × 10^−4^, respectively. Other hyper-parameters, including the batch size, weight decay, decay rate, etc, were, respectively, set to 2, 1 × 10^−4^, and 0.9. Simultaneously, the learning rate was initially set to 1 × 10^−4^ for the incipient 5 × 10^4^ iterations, then reduced to 1×10^−5^ for the subsequent 2 × 10^4^ iterations. Additionally, it was decreased by a factor of 10 at 40,000 iterations. For training, the 256 RoIs of each image were utilized. For testing, 1000 RoIs per image were utilized.

**Figure 15:**
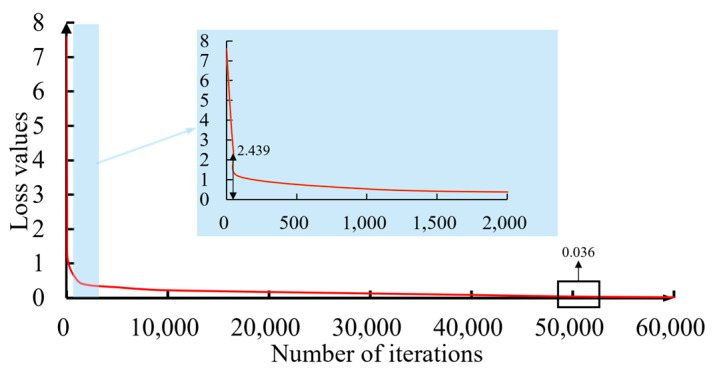
Learning curve of the training process.

During the process of training the Mask R-CNN algorithm, the loss function was monitored to assess the progress of the training. The initial loss was a considerable 7.568, but the loss experienced a swift decline to 2.439 within the first 50 iterations. After an extensive 50,000 iterations, it reached a minuscule 0.036, as shown in Figure 15. As a result, the optimal weights were acquired and saved, which means that trained algorithms had been obtained. The parameters were subjected to trial-and-error analysis through experimentation to achieve the desired performance for accurately segmenting the underlying surface marks.

### 3.5. Quantification of the Marks of the Underlying Surface

**Figure 16:**
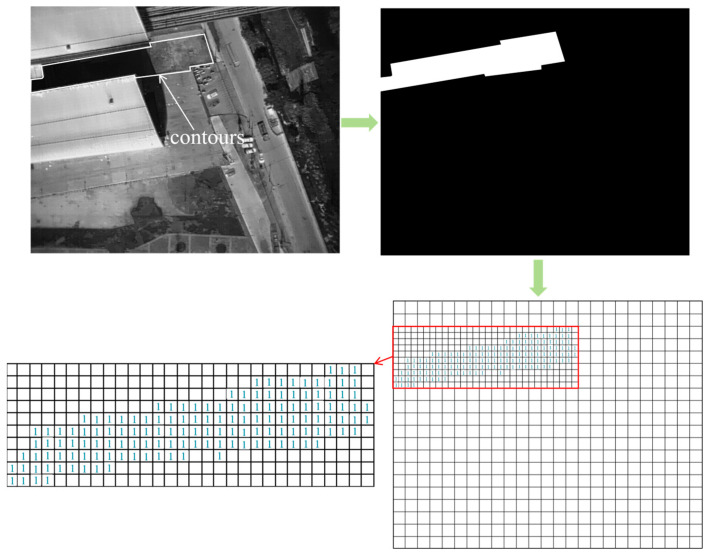
Schematic of the quantification of the underlying surface marks.

The source code of the original Mask R-CNN algorithm was added to calculate the areas of the marks of the various types of underlying surfaces at the QS URTH station. The lawn in Figure 16 was adopted as an instance to illustrate the quantification process of the marks of the underlying surfaces. At the first step, the branch of an FCN of the trained structural-optimized algorithm generates a closed polygon for the different underlying surface marks in the raw image. Then, the pixels within the polygon are assigned a value of 1, and the remaining pixels are assigned a value of 0. Thus, a binary image is generated before generating an image with the following underlying surface markings. In the final step, the geometric area of the underlying surface mark is computed by counting the total number of the pixels with the value of 1 in the generated binary image (Figure 16).

## 4. Case Evaluation

To evaluate the performance of the two Mask R-CNN algorithms according to the results of the instance segmentation of the underlying surface within the low-altitude TIR sensing images, a dataset comprising 150 new images was utilized. Furthermore, the performance of the structure-optimized algorithm was compared with that of the original algorithm using the four quantitative evaluation metrics (m*AP*, *F*_1_ score, IoU, and inference time).

### 4.1. Recognition Results

**Figure 17:**
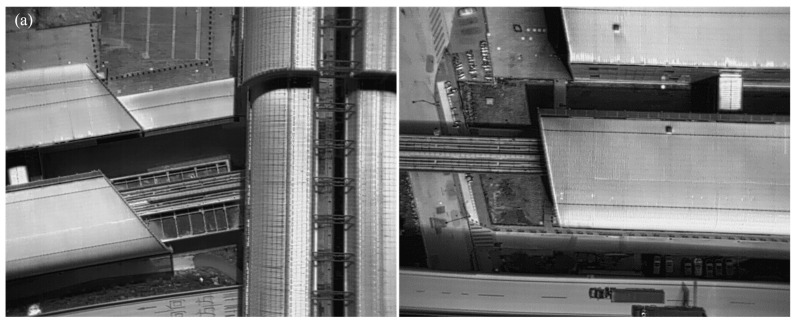

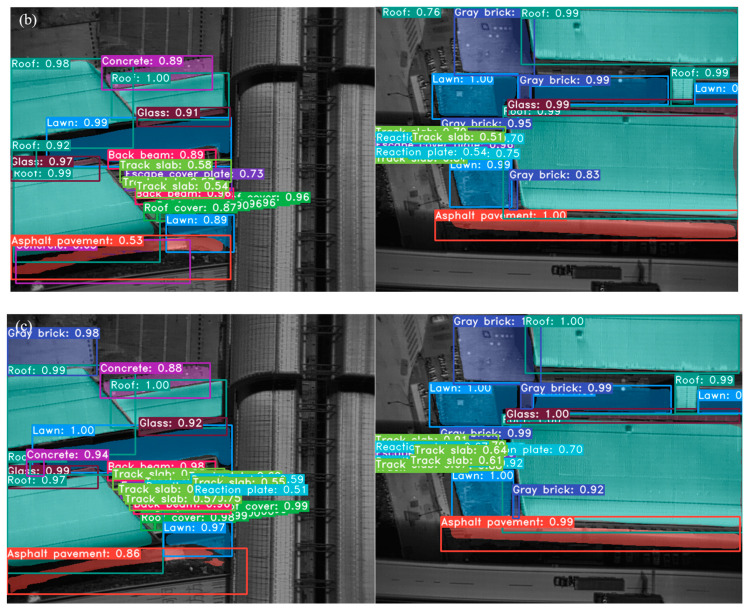
A portion of the results of the segmentation of the underlying surface marks: (**a**) raw images; (**b**) results with the original Mask R-CNN algorithm; (**c**) results with the proposed Mask R-CNN algorithm.

Based on the information provided, 150 images were input into both trained algorithms. The output of the algorithms would be a set of predicted masks for each image, indicating the regions where the underlying surface marks are present. Figure 17 displays a portion of the segmental results obtained from the new testing images.

The figures on the left side of Figure 17b,c obtained by the original Mask R-CNN algorithm have two problems: (1) For smaller targets in the figure, such as the track slab, back beam, and asphalt coating, the prediction results obtained through preliminary training are difficult to detect or cannot be detected; (2) larger underlying targets can be detected accurately, but their mask edges have wavy curves or are irregular, making it difficult to align them accurately with the contour of the underlying surface for roof and roof cover.

In comparison with the original Mask R-CNN algorithm presented by He et al. (2017) [37], the structure-optimized Mask R-CNN algorithm demonstrated improved performance by providing more pronounced and reliable information of the underlying surface masks, as shown in the figures on the right side of Figure 17b,c. The Mask R-CNN algorithms evidently furnished lucid information about the underlying surface marks. However, in contrast to the original algorithm [37], the structure-optimized algorithm outperformed because of its heightened clarity and dependability in conveying the underlying surface mask information.

### 4.2. Evaluation of the Algorithms for Instance Segmentation

A more comprehensive analysis and comparison employing quantitative evaluation metrics would serve to substantiate the superiority of the structural optimization Mask R-CNN algorithm related to the original version. Three metrics, namely the m*AP*, F_1_ score, and IoU were utilized to assess the performance of the two Mask R-CNN algorithms and the other instance segmentation approach, namely the Path Aggregation Network (PANet) [53].

To compute these metrics, the following events were defined in the image segmentation. Correctly identifying an underlying surface mark pixel was determined to be a true positive (*TP*). Conversely, a correctly identified background pixel was a true negative (*TN*). However, mistakes can occur. If a background pixel was incorrectly labelled as an underlying surface mark pixel, it was a false positive (*FP*). Similarly, missing an underlying surface mark and marking it as background resulted in a false negative (*FN*). These classifications were pivotal for calculating the accuracy metrics. The calculations of the aforementioned metrics were listed as the following formulas:

(1) $$\begin{array}{ll} mAP & \frac{TP+TN}{TP+TN+FP+FN}, \end{array}$$

(2) $$\begin{array}{ll} Precision & \frac{TP}{TP+FP}, \end{array}$$

(3) $$\begin{array}{ll} Recall & \frac{TP}{TP+FN}, \end{array}$$

(4) $$\begin{array}{ll} F_{1}\;\mathrm{score} & \frac{2}{1/Precision+1/Recall}, \end{array}$$

(5) $$\begin{array}{ll} \mathrm{IoU} & \frac{TP}{TP+FP+FN}. \end{array}$$

**Figure 18:**
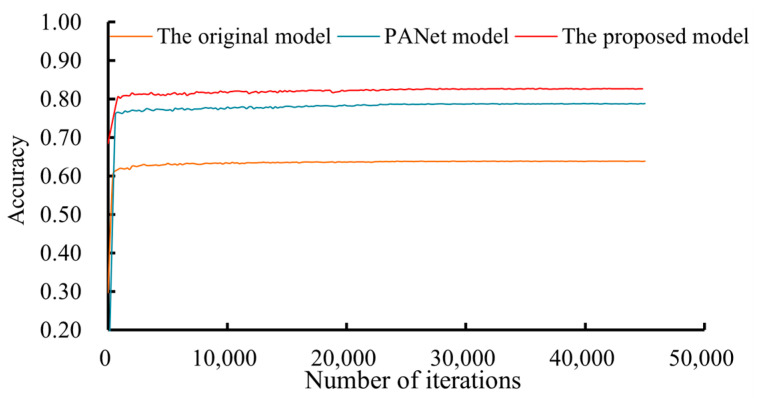
Accuracy of the instance segmentation models during the training process.

These metrics enable a quantitative assessment of the performance of the algorithms and provide insights into their accuracy, precision, and ability to perform image segmentation. For the comparison of the performance of the proposed Mask R-CNN model with other developed instance segmentation approaches, the PANet and the original Mask R-CNN model were also trained using the same training dataset. The changes in the accuracy of these models were presented in Figure 18. Figure 18 shows that both the PANet and Mask-R-CNN models converged after 25,000 iterations.

According to the values of the metrics listed in Table 3, the performance of the structural optimization algorithm surpasses that of the original algorithm for underlying surface-mark instance segmentation in low-altitude TIR sensing images of URTHs. The performance of the proposed model was also compared with those of the original model and the PANet model, as presented in Table 3. The proposed Mask R-CNN model achieved an overall accuracy of 81.5%, which was higher than those of the original model (62.6%) and the PANet model (77.8%). Similarly, the *F*_1_ score of the new method, which is crucial for understanding the balance between precision and recall, was 0.752, significantly surpassing the value of 0.552 for the original method, and 0.637 for the PANet model. The IoU, one of the most telling metrics, further illustrates the superiority of the proposed method, which achieved a remarkable value of 0.896 in contrast to the value of 0.625 of the original method, and 0.814 of the PANet model. These results underscore the advancements made by the new method, which will enable more accurate and efficient segmentation in this specific application.

### 4.3. Inference Time

Table 4 presents the inference times for the three models, considering the detection of bounding boxes, mask detection, and adding the masks to the raw image. The overall inference time holds significant importance, particularly when dealing with a substantial volume of low-altitude TIR sensing images depicting underlying surfaces.

The structure-optimized Mask R-CNN algorithm represents a substantial advancement in the domain of image segmentation, particularly for low-altitude TIR sensing images within urban rail transit hubs. It outperforms the original model and the PANet model in both speed and accuracy. Notably, the proposed model achieves a remarkable reduction in inference time (0.216 s per image) while simultaneously improving key accuracy metric compared with the original model (1.305 s per image) and the PANet model (0.473 s per image). The m*AP*, *F*_1_ score, and IoU, crucial for precise segmentation, are all substantially enhanced, being higher than those achieved by the original model and the PANet model. Herein, the ResNet-101 was used as the backbone architecture for the PANet model and the proposed model. The results obtained using PANet were almost close to those of the proposed model, although PANet had added bottom-up path augmentation, adaptive feature pooling, and fully connected fusion tricks to improve the instance segmentation performance. The proposed model was the model trained from scratch without using ImageNet pre-training weights. The segmentation results obtained using the proposed model, especially the mask m*AP*, were not better than those of PANet model. It can be concluded the ImageNet pre-training weights contribute to the improvement of instance segmentation results. These testing metrics and the contrastive analysis indicated that the obtained proposed model had a good performance for the task of instance segmentation of the underlying surface in low-altitude TIR sensing images. This compelling combination of superior accuracy and rapid processing makes the structure-optimized model an outstanding choice for the task of segmenting underlying surface marks in URTHs.

## 5. Conclusions

This paper describes a method for the instance segmentation of the underlying surface marks in low-altitude TIR sensing images. The process involves two major steps: (1) the creation of the instance segmentation dataset of the low-altitude TIR sensing images of the underlying surfaces and (2) the modification and training of the Mask R-CNN algorithm. To create a low-altitude TIR sensing image dataset, the low-altitude TIR sensing images were acquired via a low-altitude TIR sensing system. Then, annotations were drawn using LabelMe. Subsequently, the corresponding annotation file was transformed into a COCO dataset format using the Python language. With the dataset, the Mask R-CNN algorithm was structure optimized and trained to recognize the underlying surface marks. The hyperparameters suitable for specific problems were determined through case evaluation. The algorithm was then trained on the created low-altitude TIR sensing image dataset until the algorithm’s loss function reached convergence, indicating the readiness for practical applications.

For a comparative evaluation of the original Mask R-CNN algorithm, the PANet algorithm, and the structure-optimized algorithm of this study, 150 testing images were utilized. The structure-optimized Mask R-CNN demonstrated a relatively low inference time while achieving superior performance according to the m*AP*, *F*_1_ score, and IoU in comparison to those of the frequently used algorithms. The structure-optimized Mask R-CNN demonstrated enhanced precision and efficiency in identifying the underlying surface marks in the low-altitude TIR sensing images of an URTH. It is worth highlighting that the results previously discussed were obtained from a training and validation dataset encompassing a total of 1500 images. To enhance the accuracy and robustness of the method further, additional images of the other URTHs should be acquired using the low-altitude TIR sensing system. By enlarging the database, the algorithm’s performance may be improved further. Such precise classification of underlying surfaces in the TIR images promotes further advancement in the accurate calculation of the LSTs based on the low-altitude TIR remote sensing images.

## Figures and Tables

**Table 1:** Statistics on types of the underlying surfaces at QS station.

Number	Type of Underlying Surface
1	Roof
2	Lawn
3	Dutch brick pavement
4	Concrete
5	Asphalt pavement
6	Roof cover
7	Back beam
8	Reaction plate
9	Escape cover plate
10	Glass
11	Track slab

**Table 2:** Flight dates and corresponding number of the low-altitude TIR sensing images.

Date	June 2019		October 2020	November 2020	December 2020		March 2021	
	15th	16th	3rd	28th	22nd	23rd	23rd	24th
Number of the TIR sensing images	40	68	176	286	126	93	93	82

**Table 3:** Metrics of algorithms for segmentation of underlying surface marks.

Algorithm	m*AP*	*F*_1_ Score	IoU
PANet	77.8%	0.637	0.814
Original model	62.6%	0.552	0.625
Proposed model	81.5%	0.752	0.896

**Table 4:** Inference times of the algorithms for the segmentation of the underlying surface marks.

Algorithm	Inference Time (s Per Image)
PANet	0.473
Original model	1.305
Optimized model	0.216

## Data Availability

The data presented in this study are available on request from the corresponding author.
